# Selective antimicrobial potential of fucoidans against the pathogen *Listeria monocytogenes*

**DOI:** 10.3389/fmicb.2026.1731064

**Published:** 2026-03-27

**Authors:** Orla C. Cooney, Sinead T. Morrin, Rachael H. Buck, Rebecca A. Owens, Rita M. Hickey

**Affiliations:** 1 Department of Biology, Maynooth University, Maynooth, Kildare, Ireland; 2 Teagasc Food Research Centre, Moorepark, Fermoy, Cork, Ireland; 3 Abbott Nutrition, A Division of Abbott Laboratories, Columbus, OH, United States

**Keywords:** antibacterial, foodborne pathogens, fucoidan, host-pathogen interactions, internalins, *Listeria monocytogenes*, polysaccharide, seaweed

## Abstract

*Listeria monocytogenes* is a gram-positive facultative intracellular foodborne pathogen capable of causing severe infection, particularly in immunocompromised and elderly populations. Infection typically begins at the gastrointestinal mucosa, where *L. monocytogenes* employs a range of virulence factors to enable its adhesion and translocation across the intestinal epithelial barrier. *L. monocytogenes* is equipped with virulence factors known as internalins, a family of proteins which play a key role in facilitating the adhesion and invasion of host cells. In this study, we investigate the anti-adhesive and anti-invasive potential and mode of action of fucoidan derived from *Macrocystis pyrifera* and *Undaria pinnatifida* against *L. monocytogenes.* Fucoidan from *M. pyrifera* significantly inhibited adhesion of *L. monocytogenes* NCTC 5348 to HT-29 intestinal epithelial cells in a concentration-dependent manner, while fucoidan from *U. pinnatifida* showed a modest but non-significant reduction in adhesion. In contrast, both fucoidans significantly reduced bacterial invasion, resulting in reductions of 71.8 ± 9.3% and 40.8 ± 11.7%, respectively (*p* < 0.05), indicating inhibition of early infection events, likely adhesion. Further analysis of fucoidan from *M. pyrifera* against a panel of 11 additional *L. monocytogenes* strains demonstrated strong inhibitory activity against the LO28 strain, reducing adhesion by 53.0 ± 11.8% (*p* < 0.05). Interestingly, the 12 strains employed in this study had genetically diverse internalin profiles with fucoidan-susceptible LO28 possessing a similar internalin profile to NCTC 5348. This strain-specific activity observed suggests that fucoidan from *M. pyrifera* primarily acts on the bacteria to inhibit adhesion to gastrointestinal cells, and that its presence during infection is required to achieve maximum anti-adhesive efficacy. This study highlights the importance of fucoidan structure in determining bioactivity and identifies both fucoidan from *M. pyrifera* and *U. pinnatifida* as strain-specific anti-invasive ingredients against *L. monocytogenes* infection.

## Graphical Abstract

**Figure:**
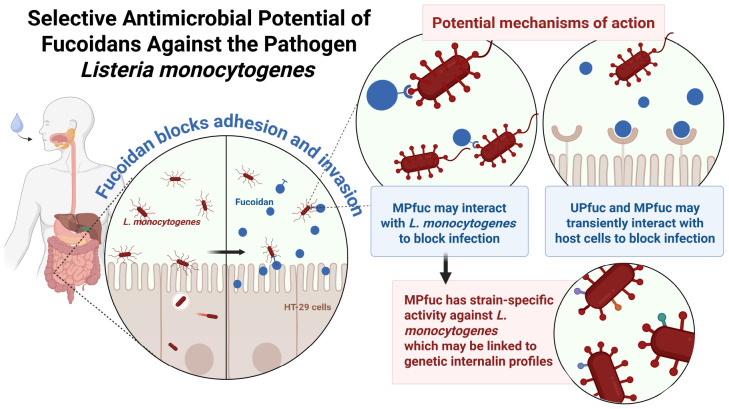
Selective antimicrobial potential of fucoidans against the pathogen *Listeria monocytogenes*. Created with Biorender.com.

## Introduction

*L. monocytogenes* is a gram-positive foodborne bacterium primarily linked to outbreaks from ready-to-eat dairy and meat products. It is a significant cause of gastrointestinal illness, manifesting as listeriosis in immunocompromised and elderly individuals, and as gastroenteritis in otherwise healthy individuals (Scallan et al., 2011). *L. monocytogenes* has evolved specialized survival mechanisms and a diverse arsenal of virulence factors such as internalins that enable it to withstand the hostile environment of the gastrointestinal tract. To establish infection, *L. monocytogenes* must effectively adhere to and invade intestinal epithelial cells while evading immune clearance by the host (Radoshevich and Cossart, 2018; Lin et al., 2024; Ireton et al., 2021). Therefore, inhibiting bacterial adherence to the host epithelial surface at an early stage of infection is critical for curbing both the incidence and severity of infection.

Despite its low incidence, *L. monocytogenes* has the highest hospitalization and case fatality rates among zoonotic diseases in the European Union (EFSA, 2023; EFSA, 2024), and while it remains largely susceptible to first-line antibiotics, emerging antimicrobial resistant strains highlight the evolving need for alternative therapies (Baquero et al., 2020; Olaimat et al., 2018). Although *L. monocytogenes* is classified into 13 serotypes, the four most common serotypes isolated from clinical, food and environmental samples are 1/2a, 1/2b, 1/2c, and 4b (Zhang et al., 2019; Braga et al., 2017; Almeida et al., 2017; Dong et al., 2023; Cavalcanti et al., 2022; Heidarlo et al., 2021; Lukinmaa et al., 2003). Bacterial adherence to host cells is often mediated by surface carbohydrates, including bacterial lipopolysaccharides and host cell glycoproteins, facilitating key interactions for host colonization (Kato and Ishiwa, 2015). This has led to extensive research exploring the use of dietary carbohydrates as soluble anti-adhesive agents which work to block this process, such as those found in milk, honey, and seaweed (Lane et al., 2019; Morrin et al., 2021; Sun et al., 2023; Li and Shah, 2014). Dietary carbohydrates offer several advantages over conventional antibiotics; they are less susceptible to antimicrobial resistance and are less likely to disrupt the gut microbiota (Becattini et al., 2016; Allen et al., 2014). *L. monocytogenes*-host interactions are frequently mediated by carbohydrates, and a number of studies have shown the potential of carbohydrates as a means of antibacterial protection against *Listeria* infection *in vitro* (Henry-Stanley et al., 2003; Chen et al., 2017; Ebersbach et al., 2010; Maganti et al., 1998; Li and Shah, 2014).

The marine environment is a particularly rich natural resource for many biologically active carbohydrates. In particular, seaweed polysaccharides have emerged as promising antimicrobials due to their structural complexity and diversity, allowing them to confer a wide range of bioactivities (Jiao et al., 2011; Lomartire and Goncalves, 2022). Among these, fucoidan, a sulfated polysaccharide derived from brown seaweed, has been extensively studied for its immunomodulatory properties (Zhang et al., 2015; Park et al., 2021). Fucoidans can generally be categorized into three structural groups: those composed of mostly 1,3- and 1,4-linked α-L-fucopyranose residues, those composed of primarily 1,3-linked α-L-fucopyranose residues, and galactofucan, which is composed of similar proportions of 1,6-linked β-L-galactopyranose and α-L-fucopyranose residues. Fucoidans are highly sulfated, with sulfate groups predominantly occurring at the C-2 and/or C-4 positions (Ale et al., 2011; Bilan et al., 2010; Lorbeer et al., 2016).

Fucoidan polysaccharides have been widely demonstrated to have broad spectrum antiviral activity by blocking viral attachment and subsequent infection (Dinesh et al., 2016; Queiroz et al., 2008; Saha et al., 2012; Hayashi et al., 2008; Sun et al., 2018; Wang et al., 2017; Jin et al., 2020; Song et al., 2020). However, studies investigating the anti-adhesive properties of fucoidans against bacterial pathogens remain comparatively limited. Existing research has primarily concentrated on the bacteriostatic and bactericidal effects of fucoidan, which does not account for anti-adhesive activity (Ayrapetyan et al., 2021; Graikini et al., 2023; Janapatla et al., 2023; Marudhupandi and Humar, 2013; Liu et al., 2019). Moreover, in the limited studies assessing anti-adherent activities, the specific mechanisms that underpin the anti-adhesive effects of fucoidan are yet to be characterized (Janapatla et al., 2023; Sun et al., 2023).

In this study, we investigated the anti-adhesive properties of two fucoidans derived from *M. pyrifera* (MPfuc) and *U. pinnatifida* (UPfuc), which possess well characterized but distinct structural features. These fucoidans are widely studied with documented bioactivity including anti-inflammatory and immunomodulatory effects (Zhang et al., 2015; Ahmad et al., 2022). In addition, previous studies have reported the monosaccharide content of these commercial fucoidans, with MPfuc having high concentrations of fucose (between 26 and 27%) and lower concentrations of other monosaccharides, while UPfuc is a galactofucan with almost equal proportions of fucose and galactose (27–28% fucose and 25% galactose). In terms of the sulfate content, reported values vary, but most studies agree that the sulfate content of MPfuc is within the range of 26–34% while UPfuc has a sulfate content within the range of 25–39% (Silva et al., 2022; Zhang et al., 2015; Vaamonde-Garcia et al., 2021). These fucoidans were examined for their ability to inhibit the attachment and invasion of *L. monocytogenes* to human HT-29 colonic epithelial cells *in vitro*. Following this, their potential modes of action were investigated, and fucoidan derived from *M. pyrifera* was screened against a panel of *L. monocytogenes* strains selected for their diverse internalin gene profiles to provide insight into the strain-specificity of MPfuc.

## Materials and methods

### Materials

Fucoidan from *Macrocystis pyrifera* (Purity > 85%, Lot #SLCF2310) and from *Undaria pinnatifida* (Purity > 95%, Lot #SLCQ0060) were purchased from Merck^®^ (St. Louis, MO, United States). The HT-29 human colonic adenocarcinoma cell line was purchased from the American Type Culture Collection (ATCC; Middlesex, United Kingdom).

### Bacterial strains and culture conditions

The bacterial strains used in this study are listed on Table 1. All strains were stored in tryptic soy broth (TSB; Becton Dickinson and Company, France) or Brain-Heart Infusion (BHI; Merck^®^) broth containing 50% (v/v) glycerol at −20°C short-term. Strains were routinely cultured from storage in TSB at 37°C in aerobic conditions.

**TABLE 1:** List of *L. monocytogenes* strains used in the anti-adhesive studies.

Strain	Serotype	Source
NCTC 5348	1/2c	National Collection of Type Cultures (NCTC; London, United Kingdom)
DPC 6895	1/2b	Dairy Products Research Centre culture collection (DPC; Teagasc Food Research Centre, Cork, Ireland)
EGDe	1/2a	Guinea pig, Cambridge, England
F2365	4b	Cheese Isolate from California Outbreak in 1985
H7858	4b	U.S Multi-State Outbreak, 1998–1999
LO28	1/2c	Clinical isolate in the faeces of a healthy pregnant woman
NCTC 11994	4b	National Collection of Type Cultures (NCTC; London, United Kingdom)
MQ110039	1/2a	Human isolate from University College Hospital (UCH) Galway
MQ110049	1/2a	Human isolate from UCH Galway
MQ120011	1/2a	Human isolate from UCH Galway
MQ120038	4b	Human isolate from UCH Galway
Scott A	4b	Clinical isolate, Massachusetts outbreak, 1983

### Mammalian cell culture conditions

HT-29 cells were routinely grown in McCoy’s 5A modified medium (Merck^®^) supplemented with 10% (v/v) fetal bovine serum (FBS; Merck^®^). Cells were maintained in 75 cm^2^ tissue culture flasks and incubated at 37°C in 5% (v/v) CO_2_ in a humidified atmosphere. Cells were passaged when the confluency reached approximately 80–90% and seeded into 12-well CellBIND^®^ plates (Corning^®^, NY, United States) at a concentration of 1 × 10^5^ cells/well. The cells were incubated at 37°C in 5% (v/v) CO_2_ in a humidified atmosphere and washed with PBS and the culture medium replaced with fresh McCoy’s 5A media (10% FBS) every second day until 100% confluent. Confluence was assessed by light microscope and typically occurred 4–6 days post-seeding.

### Cell viability assay

To ensure the polysaccharides had no effect on cell viability at the concentrations used in this study, the CellTiter^®^ 96 Aqueous One Solution Cell Proliferation Assay (Promega, Madison, WI) was performed. This colorimetric assay measures the metabolic activity of the cells by applying the MTS tetrazolium compound which is converted enzymatically by proliferating cells into a colored formazan product, to quantify the viable cells. HT-29 cells were seeded into a 96-well plate at a concentration of 1 × 10^5^ cells/well and were maintained in McCoy’s Modified Medium (10% FBS). Following overnight incubation, the spent media was aspirated and the cells were washed with phosphate buffered saline (PBS, Merck^®^). The cells were then treated with 100 μL of the polysaccharide solutions at concentrations of 25, 100 and 1000 μg/mL in phenol red-free media. The control group contained no polysaccharide. The fucoidan solutions were incubated for 1, 6 and 24 h. Following incubation with fucoidan, the cells were washed gently with PBS, exposed to 20 μL of the CellTiter^®^ 96 Aqueous One Solution reagent and incubated for 2 h at 37°C in 5% (v/v) CO_2_ in a humidified atmosphere. The absorbance of the wells was then measured at 490 nm using a 96-well microplate reader (Synergy HT BioTek, Winooski, VT, United States). Each experiment was conducted in triplicate on three separate occasions.

### Total association assays

A series of anti-adhesive assays were performed as previously described by Ross et al. (2016). The assays were performed with HT-29 cells and the *L. monocytogenes* strains in the absence (control) or presence of fucoidan on three separate occasions in triplicate. Once the HT-29 cells were 100% confluent, the bacterial strains were harvested from an overnight culture and washed twice in pre-warmed, unsupplemented McCoy’s 5A medium. The solution was then diluted to an OD_600*nm*_ corresponding to approximately 2 × 10^8^ colony forming units (CFU)/mL (Moroni et al., 2006). Prior to infecting the HT-29 cell line, the bacteria were pre-incubated for 1 h at 37°C with filter-sterilized fucoidan in pre-warmed, unsupplemented McCoy’s 5A modified medium. This gave a final concentration of 1 × 10^8^ CFU/mL bacteria for a multiplicity of infection (MOI) of 50 and a final concentration of 1, 5, 10, 25, 50, 100, 200, and 300 μg/mL of each fucoidan. To screen the different strains, a final concentration of 100 μg/mL of *M. pyrifera* fucoidan was used. Following incubation, the HT-29 cell line was washed twice in PBS. The confluent cells were then infected with 500 μL of the bacteria-fucoidan mix and incubated for 45–60 min at 37°C in 5% (v/v) CO_2_ in a humidified atmosphere. To remove any non-adherent bacteria, the HT-29 cells were washed three times with PBS and lysed with 500 μL 0.1% Triton X-100 (Merck^®^) solution in PBS. The cell lysates were then serially diluted in maximum recovery diluent (MRD, Oxoid^®^) and plated onto tryptic soy agar (TSA, Difco™). Plates were incubated overnight at 37°C and bacteria were enumerated by determining the CFU/mL.

### Bacterial growth assays

To ensure the fucoidans did not influence the growth of the bacteria during the 2 h total exposure time in the total association assays, bacterial growth assays were performed. The assay was performed as described above with some modifications: the bacterial and fucoidan mixture was incubated for 2 h, but was not applied to the cells. Following the incubation, the mixture was serially diluted in MRD and plated on TSA. The plates were incubated overnight at 37°C and bacteria were enumerated by determining the CFU/mL.

### Invasion assay

As *L. monocytogenes* is an intracellular pathogen, its ability to first adhere to and subsequently invade host cells is critical for establishing infection. Investigating both processes is essential, as inhibiting adherence can prevent infection, while blocking invasion can limit intracellular survival and dissemination to neighboring cells (Radoshevich and Cossart, 2018). To assess the ability of fucoidan to inhibit the invasion of host cells with *L. monocytogenes*, invasion assays were performed, as previously described by Marotta et al. (2014). Briefly, the assays were performed in the same manner as the total association assays described above, with some modifications: following 1 hour incubation of the fucoidan-bacterial mixture on the HT-29 cells, the mixture was aspirated. The cells were washed gently with pre-warmed gentamicin solution (100 μg/mL) in 2% FBS McCoy’s 5A media. Gentamicin was then applied to the cells and incubated for 1 h at 37°C in 5% (v/v) CO_2_ in a humidified atmosphere. Following incubation, the media was aspirated and the cells were washed gently with 2% FBS McCoy’s 5A media. Filter-sterilized 0.1% Triton X-100 was then applied to lyse the cells, the lysates were serially diluted and subsequently plated on TSA and incubated overnight at 37°C. The bacteria were enumerated by determining the CFU/mL.

### Host cell modulation assay to assess fucoidan-cell line interactions

To assess the interaction of fucoidan with the cell line, cell modulation assays were performed as per Ross et al. (2016). Upon reaching the desired confluency, the cells were washed twice with PBS and the filter-sterilized fucoidan was applied to the cell line at a concentration of 100 μg/mL. Fucoidan was incubated with the cells for 1, 2, or 24 h at 37°C in 5% (v/v) CO_2_ in a humidified atmosphere. Treated HT-29 cells were matched to their time-point control for each specific incubation time to ensure variation in the growth of the controls would not affect the interpretation of this data. Following incubation, the cells were washed five times with PBS to remove unadhered fucoidan. The bacteria were then applied and incubated at a concentration of 1 × 10^8^ CFU/mL bacteria for 45–60 min at 37°C in 5% (v/v) CO_2_ in a humidified atmosphere. Following incubation, the anti-adhesive assay was performed as described above.

### Bacterial pre-incubation assay to assess fucoidan-*L. monocytogenes* interactions

Prior to infecting the HT-29 cells, 1 × 10^8^ CFU/mL bacteria were pre-incubated with a final concentration of 100 μg/mL fucoidan for 1 h at 37°C, 5% CO_2_. Following incubation, the suspension was washed to remove residual fucoidan by centrifugation at 2,000 rpm for 7 min. The bacterial pellet was resuspended in McCoy’s 5A media, and was subsequently applied to the HT-29 cells. The remainder of the assay was performed as described above.

Following confirmation of bacterial interaction, fucoidan binding to *L. monocytogenes* was confirmed using alcian blue to detect sulfated polysaccharides in the supernatant following the centrifugation step. This method was adapted from Ramus (1977). After centrifugation, the supernatant from both control (no fucoidan) and fucoidan-treated samples was mixed with alcian blue (1 mg/mL) in 0.5 M acetic acid (pH 2.5). The mixture was incubated at room temperature for 2 h to allow complex formation. Following incubation, samples were centrifuged to pellet the alcian blue-polysaccharide complex. The absorbance of the supernatant was measured at 610 nm, and the concentration of unbound fucoidan was determined by comparison to a standard curve, with non-specific staining (control) subtracted from the total concentration in fucoidan-treated samples.

### Statistical analysis

All infection inhibition studies were carried out on at least three separate occasions in triplicate. For infection studies, data were log_10_-transformed and the unpaired student *t*-test was used to determine statistical significance. In cell viability assays, statistical difference was calculated by one-way analysis of variance (ANOVA) with Tukey’s *post-hoc* multiple comparisons test. All data were graphed using GraphPad Prism.

## Results and discussion

Fucoidans are characterized by a complex, heterogenous structure that primarily consists of fucose monosaccharides and sulfate groups. The structure of fucoidan can vary according to the molecular weight, monosaccharide composition (galactose, mannose, xylose, and glucose), uronic acids and the number of sulfate and acetyl groups (Ale et al., 2011; Bilan et al., 2010; Lorbeer et al., 2016). These structural differences influence the biological activity of fucoidan (Ale et al., 2011). In this study, two distinct fucoidans were used: fucoidan from *Macrocystis pyrifera*, which are characterized by high fucose and sulfate contents, and fucoidan from *Undaria pinnatifida*, which generally occur as galactofucans with a high sulfate content and similar proportions of galactose and fucose (Lorbeer et al., 2016; Zhang et al., 2015; Ahmad et al., 2021; Ahmad et al., 2022; Koh et al., 2019; Maruyama et al., 2015; Lee et al., 2004).

### The effect of fucoidan on the viability of HT-29 cells

**FIGURE 1:**
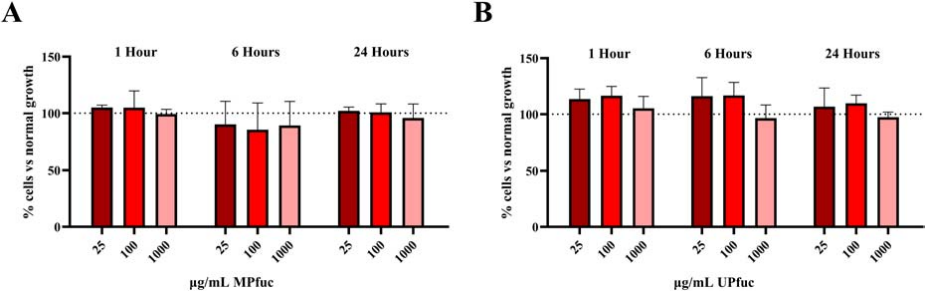
The effect of fucoidan from **(A)** *M. pyrifera* (MPfuc) and **(B)** *U. pinnatifida* (UPfuc) on HT-29 cell viability. Cells were exposed to MPfuc or UPfuc and viability was assessed by MTS assay. The data are expressed as the % of untreated cells (control, assigned as 100% cell viability, indicated by the dotted line). The data are presented as the average of biological and technical triplicates ± SD. Statistical differences were calculated by one-way ANOVA with Tukey’s multiple comparison and significance is indicated by asterisks.

Before examining the anti-adhesive activity of fucoidan, the polysaccharide was assessed for potential cytotoxic effects on HT-29 cells. Fucoidan from both *M. pyrifera* and *U. pinnatifida* did not reduce the viability of HT-29 cells at concentrations of 25, 100, and 1,000 μg/mL compared to the control (untreated, *p* > 0.05) (Figure 1). While fucoidan from other sources (particularly *F. vesiculosus)* have been shown to inhibit the proliferation of HT-29 cells, we demonstrate that the fucoidans from *M. pyrifera* and *U. pinnatifida* did not reduce the viability of HT-29 cells under these distinct conditions (Han et al., 2015; Kim et al., 2017; Bai et al., 2020b; Kim et al., 2010; de Sousa Pinheiro et al., 2017). This finding is supported by a study by Zhang et al. (2018), where the researchers reported no cytotoxic effects associated with *M. pyrifera* and *U. pinnatifida* fucoidans at the same concentrations in Caco-2 gastrointestinal cells. Several other studies reported no cytotoxic effects in other cell types including human peripheral blood mononuclear cells (PBMCs) and Vero cells (Adhikari et al., 2006; Ponce et al., 2003; Mandal et al., 2007; Dinesh et al., 2016; Trinchero et al., 2009; Lee et al., 2004; Thuy et al., 2015; Prokofjeva et al., 2013; McClure et al., 1991; Saha et al., 2012).

### Fucoidan from *M. pyrifera* has concentration-dependent anti-adhesive activity against *L. monocytogenes*

The exact antibacterial mechanisms behind the anti-adhesive properties associated with fucoidan remain unclear, and little is known about how structural differences in fucoidans influence their activity. To address this gap, we first assessed the potential of fucoidans derived from *M. pyrifera* and *U. pinnatifida* to inhibit adhesion of *L. monocytogenes* NCTC 5348 to HT-29 cells. *L. monocytogenes* NCTC 5348 was chosen due to its well-documented application in antibacterial screening assays, its broad spectrum of internalins and associated virulence determinants, and its fully sequenced genome (Murphy et al., 1996; Ismail et al., 2024; Patterson et al., 2011; Mayer et al., 2011). In addition, serotype 1/2c strains such as NCTC 5348 are prevalent isolates in clinical, environmental and food samples (Lukinmaa et al., 2003; Heidarlo et al., 2021; Gianfranceschi et al., 2003). *M. pyrifera* fucoidan (MPfuc) significantly inhibited adhesion of *L. monocytogenes* to HT-29 cells at concentrations of 50 μg/mL and above (*p* < 0.05) (Table 2). The greatest activity was observed at the highest concentration of MPfuc examined (300 μg/mL), with an 81.4 ± 2.9% decrease in adhesion (*p* < 0.05). Lower concentrations exhibited diminished efficacy, whereas concentrations above 50μg/mL produced comparable effects, indicating a plateau in activity. Fucoidan from *U. pinnatifida* (UPfuc) was also associated with a trend towards reduced bacterial adhesion, although this did not reach statistical significance (*p* > 0.05). Our study found that 100 μg/mL was the optimal concentration for MPfuc to inhibit adhesion of *L. monocytogenes*, as at higher concentrations of MPfuc, there were no significant differences in inhibition between these concentrations (*p* > 0.05). Few other studies have assessed the anti-adhesive activities of fucoidans, however, existing evidence suggests promising anti-adhesive activity. Among these, Janapatla et al. (2023) found fucoidans from *Fucus vesiculosus* and *F. serratus* reduced the adherence of *Pseudomonas aeruginosa* to Caco-2 colonic cells *in vitro* and limited bacterial colonization in a murine model (Janapatla et al., 2023). Sun et al. (2023) demonstrated fucoidan derived from *Saccharina japonica* (formerly *Laminaria japonica*) reduced the adhesion of several bacterial pathogens including *L. monocytogenes* ATCC 19115 to gastrointestinal HT-29 cells. These findings suggest that fucoidan from diverse seaweed species may exhibit anti-adhesive activity against *L. monocytogenes*, potentially due to shared structural features such as high degrees of sulfation (28.7 ± 2.6% for *S. japonica*) or a high fucose content (Silva et al., 2022; Zhang et al., 2015; Vaamonde-Garcia et al., 2021). A high degree of sulfation increases the overall negative charge of the fucoidan, which may enhance electrostatic interactions with positively charged regions of the bacterial or host cell surface. Likewise, a high fucose content may enable fucoidan to mimic host fucosylated glycans, facilitating binding to bacterial adhesins and therefore interfering with pathogen attachment.

**TABLE 2:** Concentration dependency screening of fucoidan from *M. pyrifera* and *U. pinnatifida* against *L. monocytogenes* NCTC 5348 adhesion to HT-29 cells.

Concentration (μg/mL)	*M. pyrifera* fucoidan		*U. pinnatifida* fucoidan	
	Mean % reduction ± SD^1^	*p*-value	Mean % reduction ± SD^1^	*p*-value
1	38.3 ± 7.3%	0.1849	15.9 ± 7.3%	0.4727
5	27.9 ± 23.1%	0.1524	22.4 ± 7.0%	0.4312
10	39.2 ± 3.4%	0.1039	10.3 ± 12.7%	0.6086
25	48.9 ± 37.9%	0.0545	39.9 ± 16.9%	0.5122
50	53.8 ± 30.3%	0.0319	37.5 ± 21.7%	0.5149
100	65.7 ± 20.1%	0.0037	53.1 ± 19.2%	0.3556
200	68.2 ± 22.6%	0.0371	57.2 ± 18.4%	0.3231
300	81.4 ± 2.9%	0.0158	61.1 ± 16.1%	0.2258

^1^Data are mean percentage reductions in adhesion ± standard deviation from at least three independent experiments. Statistical differences between log10-transformed treatment and control CFU/mL were assessed by student’s *t*-test.

Fucoidan is well documented to have prebiotic effects by increasing the growth of commensal bacteria in the gut (Kan et al., 2020; Shang et al., 2017; Lynch et al., 2010; Men et al., 2023; Hwang et al., 2016; Shang et al., 2016). In addition, several studies have identified fucoidan to have bacteriostatic and/or bactericidal activity against bacterial pathogens (Ayrapetyan et al., 2021; Graikini et al., 2023; Janapatla et al., 2023; Liu et al., 2017; Marudhupandi and Humar, 2013; Chua et al., 2015). To confirm the fucoidans did not influence the growth of *L. monocytogenes* NCTC 5348 during the subsequent total association assay, growth assays were performed under the same experimental conditions (Supplementary Table 1). We found that fucoidan from *M. pyrifera* and *U. pinnatifida* (25–100 μg/mL) did not influence the growth of *L. monocytogenes* NCTC 5348 compared to the control under these conditions (*p* > 0.05). This was important to confirm given that fucoidan derived from *F. vesiculosus* has been demonstrated by Poveda-Castillo et al. (2018) to inhibit the growth of *L. monocytogenes*.

### Fucoidan inhibits *L. monocytogenes* invasion

**FIGURE 2:**
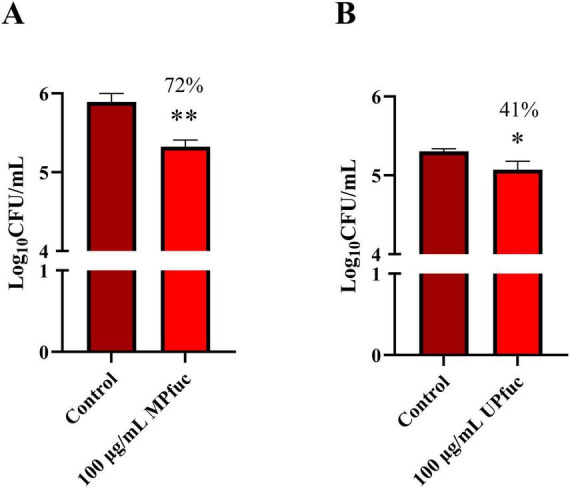
The effect of fucoidan from **(A)** *M. pyrifera* (MPfuc) and **(B)** *U. pinnatifida* (UPfuc) on invasion of *L. monocytogenes*. *L. monocytogenes* NCTC 5348 was pre-treated with MPfuc, UPfuc or the control (media, no fucoidan) and subsequently incubated with HT-29 cells to assess invasion. Data are means ± standard deviation from biological and technical triplicates. Statistical differences between treatment and control were assessed by student’s *t*-test, and significance is indicated by asterisks (*p < 0.05, **p < 0.01).

We investigated if fucoidan could limit bacterial invasion of the host cells, thus reducing infection with *L. monocytogenes* NCTC 5348. To demonstrate this, gentamicin is applied after infection which ensures any external adhered or unadhered *L. monocytogenes* is killed before selectively lysing the HT-29 cells, allowing bacterial invasion to be assessed. MPfuc significantly reduced invasion of HT-29 cells (71.8 ± 9.3%) by *L. monocytogenes* NCTC 5348 (*p* < 0.01) (Figure 2A). Fucoidan from *U. pinnatifida* also reduced invasion to a slightly lesser degree, with a 40.8 ± 11.7% reduction in invasion (*p* < 0.05) (Figure 2B). This indicates that fucoidan from both sources is likely inhibiting an early stage of infection of the cells, possibly adhesion. Comparison of the results of the adhesion and invasion assays suggests that the reduction in invasion is largely driven by decreased bacterial attachment. In relation to MPfuc, the reduction in invasion closely mirrored the reduction in adhesion (71.8% invasion versus 65.7% adhesion), and a similar relationship was observed for UPfuc (40.8% invasion vs. 53.1% adhesion), although the adhesion results were not significant. This discrepancy between the anti-adhesive and anti-invasive activities of UPfuc likely reflects a modest anti-adhesive effect that, while not statistically significant in isolation, translated into a significant downstream reduction in invasion of *L. monocytogenes.* In addition, invasion is a more specific read-out when compared to the total association assays, which measure all adhesion and invasion events that have occurred between the bacteria and the cell line. These results indicate that both fucoidans are likely acting primarily by reducing initial adhesion and the reduction in invasion is a consequence of reduced bacterial attachment, rather than inhibiting a later step in infection such as invasion or intracellular survival of the bacteria within host cells.

The differences observed between both the anti-adhesive and anti-invasive results of MPfuc and UPfuc may be partly explained by their structural differences, particularly the high galactose content of UPfuc, and the presence of 1,6 linkages (Lee et al., 2004; Hanisch, 2023). These structural features which are not present in MPfuc, may sterically hinder binding of UPfuc to *L. monocytogenes* or host cells, reducing the ability of the fucoidan to block bacterial adhesion and invasion compared to MPfuc. Although both fucoidans have comparable sulfation levels and therefore similar charges, the contrasting monosaccharide compositions and linkages are likely to influence the activity of these fucoidans (Silva et al., 2022; Zhang et al., 2015; Vaamonde-Garcia et al., 2021). These structural differences may give MPfuc an enhanced ability compared to UPfuc to mimic host fucosylated glycans, enabling stronger binding or competitive inhibition of bacterial adhesins.

### Mechanisms of action of fucoidan

**FIGURE 3:**
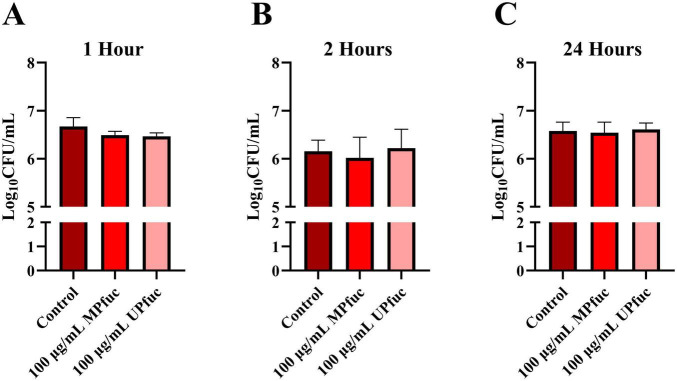
The effect of pre-treating HT-29 cells with fucoidan from *M. pyrifera* (MPfuc) and *U. pinnatifida* (UPfuc) prior to *L. monocytogenes* infection. MPfuc, UPfuc or control (media, no fucoidan) were applied to HT-29 cells for 1 **(A)**, 2 **(B)** or 24 **(C)** hours. Data are means ± standard deviation from biological and technical triplicates. Statistical differences between control and treatment were assessed by student’s *t*-test, with significance indicated by asterisks.

The inhibition of *L. monocytogenes* adhesion and invasion by fucoidans may occur through multiple mechanisms: (1) fucoidans may act as decoy receptors by binding directly to the bacteria or modulating bacterial expression of virulence factors, thereby diminishing infectivity; or (2) fucoidans may interact with or alter the expression of host gastrointestinal cell receptors, reducing bacterial adherence or invasion. To explore these potential mechanisms of action, two additional assays were conducted to evaluate the interactions between fucoidan, the bacterial cells, and the host cell line. In the cell modulation assays, fucoidan was exposed to the cell line, unbound polysaccharide was removed by washing and *L. monocytogenes* NCTC 5348 was then applied to the HT-29 cells. Fucoidan was applied for one and two hours to capture rapid or transient interactions with epithelial cell surface components, while a 24 h exposure was used to assess whether prolonged exposure induced more sustained effects on the cell line. After exposure to HT-29 cells for 1 h, MPfuc and UPfuc displayed a trend towards reducing subsequent infection by 31.9 ± 19.7 and 35.2 ± 21.3%, respectively, however these results were not significant (*p* > 0.05) (Figure 3A). Interestingly, this anti-adhesive trend was not evident in longer incubation times of two and 24 h (Figures 3B,C, respectively).

The diminished efficacy of fucoidans observed in cell modulation assays over time may be attributed to several underlying factors. Several studies have previously demonstrated that fucoidan is endocytosed by cells *in vitro* and can also be absorbed systemically following oral administration *in vivo* (Nagamine et al., 2014; Tokita et al., 2010; Kadena et al., 2018). Nagamine et al. (2014) and Zhang et al. (2018) demonstrated that addition of fucoidan to a transwell culture of Caco-2 gastrointestinal cells led to the absorption of fucoidan across the intestinal barrier, with maximal absorption observed at 60 and 120 min after administration. This may suggest that after 1–2 h of incubation, as used in this study, fucoidan is internalized by HT-29 cells, limiting its ability to inhibit the adherence of *L. monocytogenes*. However, only a small fraction of fucoidan is known to be transported across Caco-2 cells into the basolateral compartment of transwell plates (0.0076 ± 0.0003 of 100 μg/mL after 1 h), as observed by Nagamine et al. (2014), which may indicate that fucoidan may be absorbed and retained within the cells. Once internalized, fucoidan may be degraded or stored within the cell, explaining the reduced efficacy over time. This hypothesis is supported by a study by Bai et al. (2020a) whereby the researchers reported fucoidan from *F. vesiculosus* was likely transported into cells via clathrin-mediated endocytosis.

In terms of applications, fucoidan remains intact during intestinal transit and reaches the large intestine mostly intact (Tokita et al., 2010), where it may exert these anti-adhesive effects against *L. monocytogenes*. This transient effect on host cells has several implications for the use of fucoidan as an anti-infective ingredient, and higher concentrations may be needed to sustain a prolonged effect on gastrointestinal cells. Alternatively, formulation strategies such as slow-release delivery systems may enhance its utility as an anti-infective compound. Fucoidan may also have moderate binding affinity to the host cells receptors, leading to competitive inhibition of *L. monocytogenes* binding to the host cell. When the fucoidan is incubated for longer, this may result in detachment/diffusion or internalization of the fucoidan, allowing for *L. monocytogenes* to adhere and infect the cells.

**FIGURE 4:**
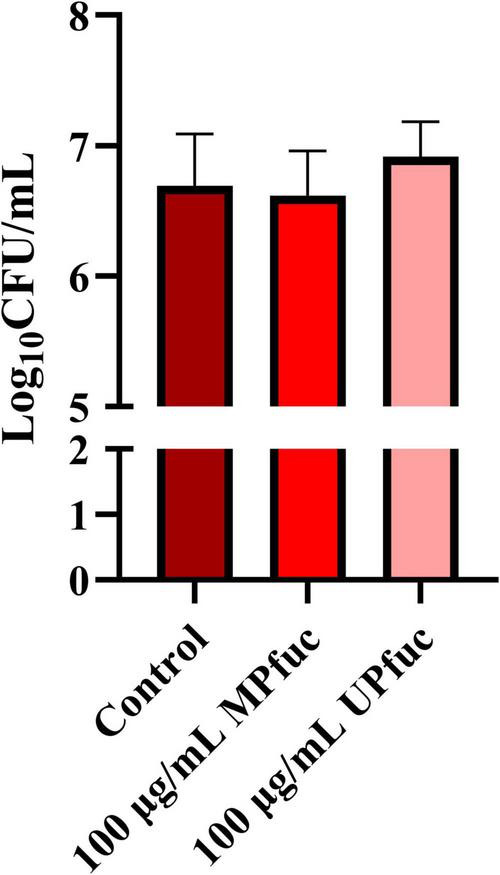
The effect of pre-treating *L. monocytogenes* with fucoidan from *M. pyrifera* (MPfuc) and *U. pinnatifida* (UPfuc) prior to infection of HT-29 cells. *L. monocytogenes* NCTC 5348 was incubated with MPfuc or UPfuc (100 μg/mL) or control (media, no fucoidan). Data are means ± standard deviation from at least biological and technical triplicates. Statistical differences between control and treatment were assessed by student’s *t*-test, with significance indicated by asterisks.

We next investigated whether a direct interaction between fucoidan and *L. monocytogenes* contributes to the observed inhibitory activity. In contrast to the total association assay (Table 2), where fucoidan is present throughout the adhesion process and can act simultaneously on both the bacteria and host cells, the bacterial pre-incubation assay isolates the bacterial component by removing unbound fucoidan prior to infection of HT-29 cells. As evident in Figure 4, pre-incubating MPfuc with *L. monocytogenes* and subsequently removing unadhered polysaccharide by centrifugation resulted in a small, non-significant reduction in bacterial adherence to gastrointestinal cells. In parallel, to explore the possibility of an interaction between fucoidan and *L. monocytogenes*, alcian blue staining was used to detect unbound fucoidan in the supernatant following centrifugation of the fucoidan-bacteria mix. Approximately 39 μg/mL of MPfuc remained unbound after centrifugation (*p* < 0.05), suggesting that approximately 61 μg/mL MPfuc had bound to *L. monocytogenes* within the pellet (Supplementary Table 2). This partial binding supports the existence of a fucoidan-bacterial interaction. The lack of a statistically significant reduction in adhesion following the wash-off step may therefore reflect several factors. Firstly, not all fucoidan bound to *L. monocytogenes*, which therefore reduces the effective concentration available to interfere with adhesion in the subsequent exposure step to host cells. Secondly, if the interaction between MPfuc and *L. monocytogenes* is a weak, transient interaction, the centrifugation step may cause dissociation of fucoidan from the bacterial cell surface, thereby reducing its inhibitory effect. In contrast, during the total association assay, fucoidan remains present throughout infection, allowing for low affinity or reversible interactions which collectively contribute to a significant reduction in adhesion.

Alternatively, fucoidan-bacterial interactions may be enhanced during active infection, for example, through binding to virulence factors or adhesins that are upregulated or expressed only under infection conditions in the presence of the host cell line. In this case, the significant reduction in adhesion would only be observed when both the bacteria and host cells are present simultaneously, as in the total association assays, and supports the conclusion that maximal anti-adhesive activity requires fucoidan to be present during the infection process. The reduction in fucoidan concentration following the wash-off step, would therefore reduce the anti-adhesive effect of fucoidan observed in the bacterial interaction assay.

These fucoidan-bacterial interactions may involve modulation of bacterial virulence factors that are only expressed during infection or, alternatively, fucoidan may function as a decoy receptor. Fucoidan has previously been shown to alter the expression of virulence factors, including adhesins AlpA and BabA of *Helicobacter pylori*, which are essential for colonizing the intestinal surface (Chen et al., 2022). Similarly, fucoidan may also be altering the expression of *L. monocytogenes* virulence factors, thereby contributing to decreased infection of HT-29 cells. However, this assay provides indirect evidence of an interaction between MPfuc and *L. monocytogenes*, and therefore confirmatory analyses are needed to verify if a potential binding interaction occurs.

Fucoidan may also mimic the fucosyl residues of mucin proteins in the gastrointestinal tract, acting as a decoy receptor to inhibit bacterial adhesion and infection. Hanisch et al. (2021) demonstrated that fucoidan from *F. vesiculosus—*along with its depolymerized forms from *F. vesiculosus* and *U. pinnatifida—*can function as decoy receptors for norovirus. These fucoidans bind to the viral lectin-like VP1 capsid protein, blocking its interaction with human gastric mucin-type O-glycans, an essential step in norovirus infection. In addition, Janapatla et al. (2023) demonstrated fucoidans bound to key virulence proteins TpsA1/CdiA2 and TpsA2/CdiA1 of *P. aeruginosa*, blocking their ability to bind host fucosylated mucins.

UPfuc did not display any anti-adhesive activity in the bacterial interaction assay under these conditions (Figure 4), which suggests that UPfuc does not strongly or stably associate with *L. monocytogenes* in the absence of host cells. However, given that UPfuc significantly reduced bacterial invasion and showed a non-significant trend toward reduced adhesion in the total association assay, this may indicate that UPfuc, like MPfuc, depends on its continuous presence during infection to exert its activity against *L. monocytogenes*. However, as UPfuc did not significantly reduce adhesion in total association assays, only MPfuc was used for subsequent screening of anti-adhesive activity against other *L. monocytogenes* strains using total association assays.

### Screening fucoidan from *Macrocystis pyrifera* for anti-adhesive activity against a bank of *Listeria monocytogenes* strains

**FIGURE 5:**
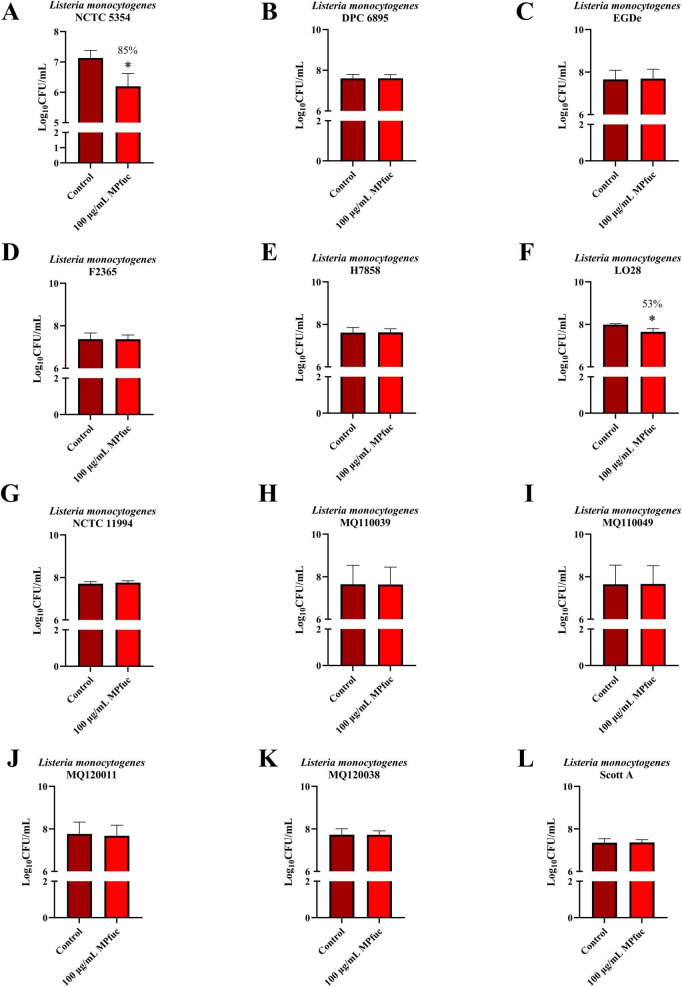
The anti-adhesive effect of fucoidan derived from *M. pyrifera* (MPfuc) on HT-29 cells infected with 12 *Listeria monocytogenes* strains. MPfuc (100 μg/mL) or control (media, no fucoidan) were screened for anti-adhesive activity against: NCTC 5348 **(A)**, DPC 6895 **(B)**, EGDe **(C)**, F2365 **(D)**, H7858 **(E)**, LO28 **(F)**, NCTC 11994 **(G)**, MQ110039 **(H)**, MQ110049 **(I)**, MQ120011 **(J)**, MQ120038 **(K)**, and Scott A **(L)**. Data are means ± standard deviation from at least biological and technical triplicates. Statistical differences between control and treatment were assessed by student’s *t*-test, with significance indicated by asterisks (*p < 0.05).

MPfuc was next assessed for the ability to inhibit the adhesion of a bank of *L. monocytogenes* strains with diverse genetic internalin profiles. Internalins are key virulence proteins found in *L. monocytogenes* that are characterized by leucine-rich repeat (LRR) domains that mediate the interactions between the bacteria and ligands on the host cell surface. These proteins can be secreted or cell-bound, and are involved in several processes including adhesion and invasion of host cells, cell-to-cell spread, host immune evasion, biofilm formation and virulence in different cell types (Burdová et al., 2024; Popowska et al., 2017; Balandyté et al., 2011; Ghosh et al., 2018; Ling et al., 2021; Ireton et al., 2021; Sabet et al., 2008; Raffelsbauer et al., 1998). By screening a panel of 12 *L. monocytogenes* strains, potential correlations between fucoidan activity and the genetic internalin profiles of the strains were investigated. Fucoidan from *M. pyrifera* displayed a strain-specific effect in total association assays, with anti-adhesive activity against only two strains of *L. monocytogenes* - NCTC 5348 and LO28 (Figure 5). Fucoidan from *M. pyrifera* reduced LO28 adhesion to HT-29 cells by 53.0 ± 11.8% (*p* < 0.05). This strain-specific effect against *L. monocytogenes* indicates that MPfuc is likely acting primarily on the bacteria to inhibit adhesion, rather than affecting the host cell surface which would be associated with a broader inhibition across the strains. Based on the annotated genomes of these strains, we compared the internalin profiles of fucoidan-susceptible and non-susceptible strains and found that susceptibility may be linked to the presence of specific bacterial internalin genes (Supplementary Table 3). Fucoidan may act by binding to or interfering with the expression or function of these strain-specific internalin genes. However, while we found that both fucoidan-susceptible strains NCTC 5348 and LO28 share the same genetic internalin profile, one non-susceptible strain, EGDe, also shares the same internalin profile. As these profiles are based solely on genomic data, they do not indicate which internalins are actively expressed during gastrointestinal infection, and therefore further research is warranted to investigate the expression of these genes and to validate the functional role in fucoidan sensitivity. Furthermore, the observed strain-specific differences may reflect other differences, specifically in cell surface components such as teichoic acid or exopolysaccharides, which may alter the surface charge and adhesion of *L. monocytogenes.* Internalins that may play a role in the strain-specific susceptibility include InlH, which plays an important role in the virulence of strains encoding this gene (Raffelsbauer et al., 1998) and InlL, which binds to the fucosylated mucin Muc2, which structurally resembles fucoidan (Popowska et al., 2017). Fucoidan may disrupt adhesion by binding to or altering the expression of these internalin proteins, subsequently blocking any interactions between *L. monocytogenes* and the cell line. These observations suggest that the presence of specific internalins, particularly those capable of binding fucosylated glycans, may influence the susceptibility of a strain to fucoidan. InlJ, is also present in susceptible strains and binds fucosylated mucins, but is not expressed *in vitro* during infection of HT-29 cells, so is unlikely to play a role in susceptibility to fucoidan (Sabet et al., 2008; Lindén et al., 2008).

The results show that fucoidan represents a novel ingredient with highly specific anti-adhesive activity against certain strains of *L. monocytogenes.* Fucoidan has several advantages over conventional antibiotics, as it can promote immunomodulation and the growth of beneficial bacteria in the gut, including beneficial *Lactobacillus* and *Bifidobacterium* species (Zhang et al., 2015; Hwang et al., 2016; Kan et al., 2020; Shang et al., 2016). Fucoidan has also demonstrated its safety in humans and represents a sustainable and environmentally friendly source of bioactive polysaccharides (Tokita et al., 2010; Kadena et al., 2018; Lomartire and Goncalves, 2022). Interestingly, in a recent study, fucoidan was identified as a promising environmental antimicrobial agent for mitigating *L. monocytogenes* biofilm in seafood handling environments, again highlighting its potential in enhancing food safety (Roy et al., 2025). Future studies should delve deeper into the mechanisms of action underlying the effects of fucoidan on *L. monocytogenes* NCTC 5348 and LO28 strains, and to determine if this strain-specific anti-adhesive activity translates to *in vivo* settings. This study highlights how the inherent structural variability of fucoidan can greatly influence its biological activity. Various factors such as the seaweed species, and the extraction and purification methods all contribute to this variability, which certainly limits and complicates direct comparison between fucoidans and poses challenges for their development as anti-infective ingredients (Sun et al., 2023; Yuan and Macquarrie, 2015).

## Conclusion

We have demonstrated in this study that fucoidans from two species, *M. pyrifera* and *U. pinnatifida* have anti-invasive activity against *L. monocytogenes.* We propose that the structural differences in the fucoidans translate to different degrees of anti-infectivity, and that fucoidans act through preventing the adhesion and subsequent invasion of *L. monocytogenes*. Although the exact mechanism underlying this activity remains unknown, we show that the fucoidans likely interact with the bacteria during infection to exert a protective effect against *L. monocytogenes* infection. These findings suggest that fucoidans could be effective and promising candidates against *L. monocytogenes* infection. Only through further understanding of the structure and mode of action of fucoidans can such polysaccharides reach their potential as anti-infective ingredients. Beyond this antimicrobial potential, fucoidan’s well-documented safety, prebiotic and immunomodulatory activity make it an excellent candidate as a functional food ingredient.

## Data Availability

The original contributions presented in this study are included in this article/Supplementary material, further inquiries can be directed to the corresponding author.
