# Supplementary material for: Selective antimicrobial potential of fucoidans against the pathogen Listeria monocytogenes
Source: Front Microbiol. 2026 Mar 27;17:1731064. doi: 10.3389/fmicb.2026.1731064 (PMC13067252; doi:10.3389/fmicb.2026.1731064)
Supplement: Supplementary file 1 [file Table_1.docx]

**Supplementary Table 1. The effect of fucoidan from** *M. pyrifera* **(MPfuc) and** *U. pinnatifida (***UPfuc) on the growth of *L. monocytogenes* strains NCTC 5348 and LO28.**

| **Strain:** | **NCTC 5348** | | **lo28** |
| --- | --- | --- | --- |
|  | **MPfuc** | **UPfuc** | **MPfuc** |
| 25 µg/mL | N/D | N/D |  |
| 50 µg/mL | N/D | N/D |  |
| 100 µg/mL | N/D | N/D | N/D |

Control was media with no fucoidan. Data are from biological duplicate and technical triplicates. Statistical differences between control and treatment were assessed by student’s t-test with significance indicated by asterisks. N/D = no statistical difference determined. Blank cell = not tested.

**Supplementary Table 2. Alcian blue staining of fucoidan from** *M. pyrifera* **(MPfuc) binding to *L. monocytogenes*.**

|  | Fucoidan |
| --- | --- |
| MPfuc in supernatant (unbound) | **39.3 ± 10.5 %*** |
| MPfuc in pellet (bound) | **60.6 ± 10.5 %*** |

The concentration of unbound fucoidan was determined by comparison to a standard curve. Data represent the mean of three biological and three technical replicates. Statistical difference between control and treatment were assessed by student’s t-test (**p* < 0.05).

**Supplementary Table 3. The distribution of internalin genes among *L. monocytogenes* strains used in this study.**

| INTERNALIN (inl) | NCTC 5348 | LO28 | EGDe | MQ120011 | MQ110049 | MQ110039 | ScottA | F2365 | H7858 | NCTC 11994 | DPC 6895 | MQ120038 |
| --- | --- | --- | --- | --- | --- | --- | --- | --- | --- | --- | --- | --- |
| LINEAGE | **I** | **II** | **II** | **II** | **II** | **II** | **I** | **I** | **I** | **I** | **I** | **I** |
| SEROTYPE | **2** | **1/2c** | **1/2a** | **1/2a** | **1/2a** | **1/2a** | **4b** | **4b** | **4b** | **4b** | **1/2b** | **4b** |
| internalin H (inlH) | + | + | + |  |  |  |  |  |  |  |  |  |
| inlH/inlC2 family class 1 inl (inlH) |  |  |  |  |  | + | + | + | + | + | + | + |
| inlC2 |  |  |  | + | + |  |  |  |  |  |  |  |
| inlA | + | + | + | + | + | + | + | + | + | + | + | + |
| GW domain-containing class 2 internalin inlB | + | + | + | + | + | + | + |  | + | + | + |  |
| inlB B-repeat-containing protein |  |  |  |  |  |  | + | + | + | + |  |  |
| inlC | + | + | + | + | + | + | + | + |  | + | + | + |
| inlD |  |  |  | + | + | + |  |  |  |  |  |  |
| inlE | + | + | + | + | + | + |  |  |  |  |  |  |
| inlF | + | + | + | + | + |  |  |  |  |  |  |  |
| inlG | + | + | + | + | + |  |  |  |  |  |  |  |
| inlI | + | + | + | + | + | + | + | + | + | + | + |  |
| inlJ | + | + | + | + | + |  |  |  |  |  |  |  |
| inlK | + | + | + | + | + | + | + | + |  | + | + | + |
| inlL | + | + | + | + | + |  |  |  |  |  |  |  |
| inlP | + | + | + | + | + |  | + | + | + | + | + | + |
| internalin N-terminal domain containing protein |  |  |  |  |  |  | + | + |  | + | + |  |
| lmo0171 family class 1 inl | + | + | + | + | + | + |  |  |  |  |  |  |
| lmo0331 family class 1 inl | + | + | + | + | + |  |  |  |  |  |  |  |
| lmo0514 family class 1 inl | + | + | + | + | + | + | + | + | + |  | + | + |
| lmo0549 family Wxl domain-containing class 2 internalin | + | + | + | + | + | + |  |  |  |  |  |  |
| lmo0610 family class 1 inl | + | + | + | + | + | + |  |  |  |  |  |  |
| lmo0801 family class 1 inl | + | + | + |  |  | + |  |  |  |  |  |  |
| lmo1136 family class 1 inl | + | + | + |  |  | + |  |  |  |  |  |  |
| lmo1289 family class 1 inl | + | + | + |  |  | + |  |  |  |  |  |  |
| lmo2027 family class 3 inl | + | + | + | + | + |  |  |  |  |  |  |  |
| lmo2396 family class 3 inl | + | + | + | + | + |  |  |  |  |  |  |  |
| lmo2445 family class 3 inl | + | + | + | + | + |  | + | + | + | + | + | + |
| 3 x inlB B-repeat containing protein |  |  |  |  |  |  | + |  |  |  |  |  |
| 4 x inlB B-repeat containing protein |  |  |  |  |  |  |  |  | + | + | + |  |

The presence of a gene in a strain’s genome is indicated by a plus (+) symbol. Data were obtained from the annotated genomes published on Genbank, Refseq OR through NCTC annotated genome (NCTC 5348 only). If Refseq annotated genomes were available, these were used preferentially over Genbank annotated genomes.
